# 2-Amino-5-(1*H*-tetra­zol-5-yl)pyridinium chloride

**DOI:** 10.1107/S1600536808031164

**Published:** 2008-10-31

**Authors:** Jing Dai, Xiao-Chun Wen

**Affiliations:** a Ordered Matter Science Research Center, College of Chemistry and Chemical Engineering, Southeast University, Nanjing 210096, People’s Republic of China

## Abstract

In the title salt, C_6_H_7_N_6_^+^·Cl^−^, there are two organic cations with similar conformations and two chloride anions in the asymmetric unit. The pyridine and tetra­zole rings are essentially coplanar in each cation, with dihedral angles of 4.94 (15) and 5.41 (14)°. The pyridine N atoms are protonated. The crystal packing is stabilized by N—H⋯N and N—H⋯Cl hydrogen bonds, forming an infinite sheets parallel to the (101).

## Related literature

For uses of tetra­zole derivatives, see: Dai & Fu (2008 ▶); Wang *et al.* (2005 ▶); Wen (2008 ▶); Xiong *et al.* (2002 ▶).
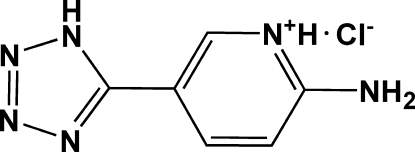


## Experimental

### 

#### Crystal data

C_6_H_7_N_6_^+^·Cl^−^
*M* *_r_* = 198.63
Monoclinic, 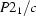

*a* = 14.043 (3) Å
*b* = 13.238 (3) Å
*c* = 9.5766 (19) Å
β = 109.35 (3)°
*V* = 1679.7 (6) Å^3^
*Z* = 8
Mo *K*α radiation
μ = 0.41 mm^−1^
*T* = 298 (2) K
0.45 × 0.25 × 0.20 mm

#### Data collection

Rigaku Mercury2 diffractometer
Absorption correction: multi-scan (*CrystalClear*; Rigaku, 2005 ▶) *T*_min_ = 0.859, *T*_max_ = 0.920
16820 measured reflections
3855 independent reflections
3123 reflections with *I* > 2σ(*I*)
*R*_int_ = 0.034

#### Refinement

*R*[*F* ^2^ > 2σ(*F* ^2^)] = 0.048
*wR*(*F* ^2^) = 0.125
*S* = 1.11
3855 reflections
235 parameters
H-atom parameters constrained
Δρ_max_ = 0.28 e Å^−3^
Δρ_min_ = −0.23 e Å^−3^

### 

Data collection: *CrystalClear* (Rigaku, 2005 ▶); cell refinement: *CrystalClear*; data reduction: *CrystalClear*; program(s) used to solve structure: *SHELXS97* (Sheldrick, 2008 ▶); program(s) used to refine structure: *SHELXL97* (Sheldrick, 2008 ▶); molecular graphics: *SHELXTL* (Sheldrick, 2008 ▶); software used to prepare material for publication: *SHELXTL*.

## Supplementary Material

Crystal structure: contains datablocks I, global. DOI: 10.1107/S1600536808031164/bh2196sup1.cif

Structure factors: contains datablocks I. DOI: 10.1107/S1600536808031164/bh2196Isup2.hkl

Additional supplementary materials:  crystallographic information; 3D view; checkCIF report

## Figures and Tables

**Table 1:** Hydrogen-bond geometry (Å, °)

*D*—H⋯*A*	*D*—H	H⋯*A*	*D*⋯*A*	*D*—H⋯*A*
N1—H1⋯N5^i^	0.86	2.59	3.317 (2)	143
N1—H1⋯N6^i^	0.86	2.04	2.894 (2)	171
N2—H2*A*⋯N5^i^	0.86	2.15	2.972 (3)	160
N7—H7⋯N11^ii^	0.86	2.57	3.304 (2)	144
N7—H7⋯N12^ii^	0.86	2.06	2.913 (2)	169
N8—H8*A*⋯N11^ii^	0.86	2.20	3.024 (3)	160
N2—H2*B*⋯Cl1^iii^	0.86	2.36	3.2187 (18)	177
N3—H3*A*⋯Cl2^i^	0.86	2.17	3.0047 (19)	165
N8—H8*B*⋯Cl2^iv^	0.86	2.39	3.2432 (18)	172
N9—H9*A*⋯Cl1^v^	0.86	2.18	3.0031 (18)	160

Symmetry codes: (i) 
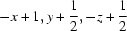
; (ii) 
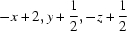
; (iii) 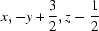; (iv) 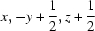; (v) 
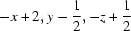
.

**Fig. 1.**
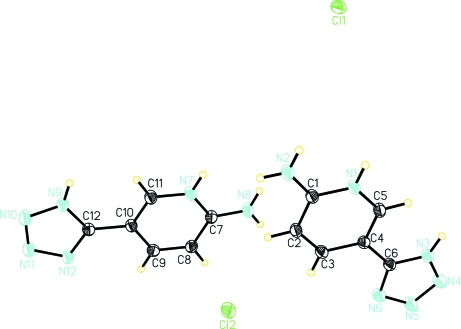
A view of the title compound with the atomic numbering scheme. Displacement ellipsoids were drawn at the 30% probability level.

**Fig. 2.**
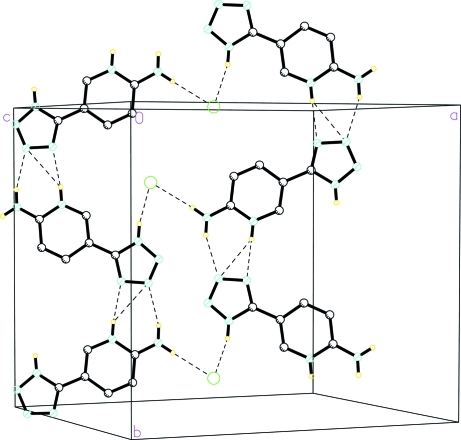
The crystal packing of the title compound viewed along the c axis, showing the two-dimensional hydrogen bonds network. H atoms not involved in hydrogen bonding (dashed lines) have been omitted for clarity.

## supplementary crystallographic information

### Comment

Tetrazole derivatives have found a wide range of applications in coordination chemistry because of their multiple coordination modes as ligands to metal ions, and for the construction of novel metal-organic frameworks (Wang *et al.*, 2005; Xiong *et al.*, 2002; Wen, 2008; Dai & Fu, 2008). We report here the crystal structure of the title salt (Fig. 1).

The title compound contains two organic cations with similar conformation and two Cl^-^ ions in the asymmetric unit. The pyridine N atoms are protonated. The pyridine rings and tetrazole rings are nearly coplanar and are twisted from each other by a dihedral angle of only 4.94 (15) and 5.41 (14)°. The packing of ions is stabilized by N—H···N and N—H···Cl hydrogen bonds, to form an infinite two-dimensional layers parallel to the (1 0 1) plane in the crystal (Table 1 and Fig. 2).

### Experimental

2-Amino-5-cyanopyridine (30 mmol), NaN _3_ (45 mmol), NH_4_Cl (33 mmol) and DMF (50 ml) were mixed in a flask under nitrogen atmosphere, and the mixture stirred at 383 K for 20 h. The resulting solution was then poured into ice-water (100 ml), and a white solid was obtained after adding hydrochloric acid (6 *M*) until pH=6. The precipitate was filtered and washed with distilled water. Colourless block-shaped crystals suitable for X-ray analysis were obtained from the crude product by slow evaporation of an ethanol/hydrochloric acid (50:1 *v*/*v*) solution.

### Refinement

All H atoms were fixed geometrically and treated as riding to their carrier atoms, with C—H = 0.93 Å (aromatic) and N—H = 0.86 Å, and with *U*_iso_(H) = 1.2*U*_eq_(carrier atom).

### Crystal data

**Table:**

C_6_H_7_N_6_^+^·Cl^−^	*F*(000) = 816
*M**_r_* = 198.63	*D*_x_ = 1.571 Mg m^−^^3^
Monoclinic, *P*2_1_/*c*	Mo *K*α radiation, λ = 0.71073 Å
Hall symbol: -P 2ybc	Cell parameters from 3690 reflections
*a* = 14.043 (3) Å	θ = 2.3–27.5°
*b* = 13.238 (3) Å	µ = 0.41 mm^−^^1^
*c* = 9.5766 (19) Å	*T* = 298 K
β = 109.35 (3)°	Block, colorless
*V* = 1679.7 (6) Å^3^	0.45 × 0.25 × 0.20 mm
*Z* = 8	

### Data collection

**Table:**

Rigaku Mercury2 diffractometer	3855 independent reflections
Radiation source: fine-focus sealed tube	3123 reflections with *I* > 2σ(*I*)
graphite	*R*_int_ = 0.034
Detector resolution: 13.6612 pixels mm^-1^	θ_max_ = 27.5°, θ_min_ = 2.7°
ω scans	*h* = −18→18
Absorption correction: multi-scan (CrystalClear; Rigaku, 2005)	*k* = −17→17
*T*_min_ = 0.859, *T*_max_ = 0.920	*l* = −12→12
16820 measured reflections	

### Refinement

**Table:**

Refinement on *F*^2^	Primary atom site location: structure-invariant direct methods
Least-squares matrix: full	Secondary atom site location: difference Fourier map
*R*[*F*^2^ > 2σ(*F*^2^)] = 0.048	Hydrogen site location: inferred from neighbouring sites
*wR*(*F*^2^) = 0.125	H-atom parameters constrained
*S* = 1.12	*w* = 1/[σ^2^(*F*_o_^2^) + (0.0556*P*)^2^ + 0.4684*P*] where *P* = (*F*_o_^2^ + 2*F*_c_^2^)/3
3855 reflections	(Δ/σ)_max_ < 0.001
235 parameters	Δρ_max_ = 0.28 e Å^−^^3^
0 restraints	Δρ_min_ = −0.23 e Å^−^^3^

### Fractional atomic coordinates and isotropic or equivalent isotropic displacement parameters (Å^2^)

**Table:**

	*x*	*y*	*z*	*U*_iso_*/*U*_eq_	
N1	0.56207 (12)	0.65987 (12)	0.17350 (18)	0.0344 (4)	
H1	0.5639	0.7245	0.1662	0.041*	
N3	0.36346 (13)	0.52444 (13)	0.37792 (19)	0.0368 (4)	
H3A	0.3600	0.5892	0.3818	0.044*	
C1	0.62505 (14)	0.60404 (14)	0.1236 (2)	0.0313 (4)	
N2	0.68458 (13)	0.64965 (13)	0.0611 (2)	0.0413 (4)	
H2A	0.6831	0.7143	0.0523	0.050*	
H2B	0.7247	0.6146	0.0292	0.050*	
N6	0.40734 (13)	0.37577 (12)	0.33322 (19)	0.0382 (4)	
N4	0.31050 (14)	0.45963 (13)	0.4305 (2)	0.0428 (4)	
C6	0.42266 (14)	0.47298 (14)	0.3184 (2)	0.0310 (4)	
N5	0.33724 (14)	0.37053 (13)	0.4030 (2)	0.0430 (4)	
C4	0.49237 (14)	0.51714 (13)	0.2516 (2)	0.0304 (4)	
C2	0.62316 (14)	0.49827 (14)	0.1406 (2)	0.0333 (4)	
H2	0.6657	0.4574	0.1086	0.040*	
C3	0.55896 (15)	0.45611 (14)	0.2039 (2)	0.0336 (4)	
H3	0.5587	0.3864	0.2160	0.040*	
C5	0.49636 (15)	0.61909 (14)	0.2344 (2)	0.0337 (4)	
H5	0.4537	0.6610	0.2646	0.040*	
N7	0.92034 (13)	0.39582 (12)	0.30105 (18)	0.0364 (4)	
H7	0.9131	0.4604	0.2998	0.044*	
N9	1.13591 (12)	0.26242 (13)	0.12521 (19)	0.0367 (4)	
H9A	1.1409	0.3272	0.1246	0.044*	
N8	0.80738 (13)	0.38416 (13)	0.4302 (2)	0.0422 (4)	
H8A	0.8043	0.4490	0.4319	0.051*	
H8B	0.7726	0.3485	0.4708	0.051*	
C7	0.86565 (14)	0.33931 (15)	0.3650 (2)	0.0329 (4)	
N10	1.19013 (14)	0.19768 (13)	0.0747 (2)	0.0425 (4)	
N12	1.08698 (13)	0.11357 (12)	0.15986 (19)	0.0374 (4)	
C12	1.07262 (14)	0.21051 (14)	0.1769 (2)	0.0311 (4)	
N11	1.16049 (14)	0.10833 (13)	0.0959 (2)	0.0429 (4)	
C10	1.00037 (14)	0.25408 (14)	0.2397 (2)	0.0304 (4)	
C9	0.94263 (15)	0.19202 (14)	0.3023 (2)	0.0339 (4)	
H9	0.9504	0.1222	0.3025	0.041*	
C11	0.98593 (16)	0.35597 (14)	0.2389 (2)	0.0363 (4)	
H11	1.0211	0.3983	0.1957	0.044*	
C8	0.87624 (15)	0.23312 (15)	0.3618 (2)	0.0337 (4)	
H8	0.8378	0.1915	0.4004	0.040*	
Cl1	0.84188 (4)	0.97883 (4)	0.45292 (7)	0.04632 (17)	
Cl2	0.65611 (4)	0.24380 (4)	0.05567 (6)	0.04478 (17)	

### Atomic displacement parameters (Å^2^)

**Table:**

	*U*^11^	*U*^22^	*U*^33^	*U*^12^	*U*^13^	*U*^23^
N1	0.0407 (9)	0.0214 (8)	0.0500 (10)	0.0034 (7)	0.0270 (8)	0.0030 (7)
N3	0.0440 (10)	0.0261 (8)	0.0491 (10)	−0.0020 (7)	0.0273 (8)	−0.0008 (7)
C1	0.0332 (10)	0.0274 (9)	0.0362 (10)	0.0024 (7)	0.0157 (8)	0.0009 (7)
N2	0.0483 (11)	0.0305 (9)	0.0589 (11)	0.0027 (7)	0.0363 (9)	0.0028 (8)
N6	0.0456 (10)	0.0267 (8)	0.0507 (10)	−0.0021 (7)	0.0270 (8)	0.0009 (7)
N4	0.0518 (11)	0.0327 (9)	0.0561 (11)	−0.0061 (8)	0.0343 (9)	−0.0012 (8)
C6	0.0334 (10)	0.0265 (9)	0.0358 (10)	−0.0001 (7)	0.0152 (8)	−0.0004 (7)
N5	0.0529 (11)	0.0299 (9)	0.0581 (11)	−0.0034 (8)	0.0346 (9)	−0.0008 (8)
C4	0.0313 (9)	0.0259 (9)	0.0368 (10)	−0.0001 (7)	0.0149 (8)	0.0011 (7)
C2	0.0341 (10)	0.0284 (9)	0.0419 (11)	0.0044 (8)	0.0186 (8)	−0.0002 (8)
C3	0.0368 (10)	0.0238 (9)	0.0422 (11)	0.0017 (7)	0.0158 (8)	−0.0001 (8)
C5	0.0358 (10)	0.0281 (9)	0.0437 (11)	0.0035 (8)	0.0218 (8)	0.0018 (8)
N7	0.0468 (10)	0.0227 (8)	0.0486 (10)	0.0051 (7)	0.0279 (8)	0.0046 (7)
N9	0.0410 (10)	0.0264 (8)	0.0510 (10)	0.0036 (7)	0.0265 (8)	0.0027 (7)
N8	0.0473 (11)	0.0334 (9)	0.0586 (11)	0.0043 (8)	0.0345 (9)	0.0034 (8)
C7	0.0328 (10)	0.0327 (10)	0.0353 (10)	0.0024 (8)	0.0143 (8)	0.0018 (8)
N10	0.0473 (11)	0.0343 (9)	0.0569 (11)	0.0066 (8)	0.0320 (9)	0.0036 (8)
N12	0.0431 (10)	0.0293 (8)	0.0477 (10)	0.0038 (7)	0.0256 (8)	0.0015 (7)
C12	0.0348 (10)	0.0266 (9)	0.0339 (9)	0.0021 (7)	0.0142 (8)	0.0033 (7)
N11	0.0489 (11)	0.0344 (10)	0.0560 (11)	0.0059 (8)	0.0316 (9)	0.0010 (8)
C10	0.0328 (10)	0.0282 (9)	0.0325 (9)	0.0027 (7)	0.0139 (8)	0.0015 (7)
C9	0.0375 (10)	0.0236 (9)	0.0431 (11)	0.0009 (7)	0.0168 (9)	0.0031 (8)
C11	0.0427 (11)	0.0296 (10)	0.0446 (11)	0.0017 (8)	0.0253 (9)	0.0037 (8)
C8	0.0344 (10)	0.0298 (10)	0.0409 (10)	−0.0010 (8)	0.0178 (8)	0.0040 (8)
Cl1	0.0567 (3)	0.0300 (3)	0.0626 (4)	−0.0067 (2)	0.0336 (3)	−0.0052 (2)
Cl2	0.0502 (3)	0.0282 (3)	0.0624 (4)	0.0046 (2)	0.0273 (3)	0.0040 (2)

### Geometric parameters (Å, °)

**Table:**

N1—C1	1.355 (2)	N7—C7	1.355 (2)
N1—C5	1.356 (2)	N7—C11	1.358 (2)
N1—H1	0.8600	N7—H7	0.8600
N3—N4	1.338 (2)	N9—N10	1.339 (2)
N3—C6	1.340 (2)	N9—C12	1.341 (2)
N3—H3A	0.8600	N9—H9A	0.8600
C1—N2	1.325 (2)	N8—C7	1.323 (2)
C1—C2	1.411 (3)	N8—H8A	0.8600
N2—H2A	0.8600	N8—H8B	0.8600
N2—H2B	0.8600	C7—C8	1.415 (3)
N6—C6	1.320 (2)	N10—N11	1.292 (2)
N6—N5	1.362 (2)	N12—C12	1.318 (2)
N4—N5	1.291 (2)	N12—N11	1.365 (2)
C6—C4	1.457 (2)	C12—C10	1.459 (2)
C4—C5	1.363 (3)	C10—C11	1.364 (3)
C4—C3	1.421 (3)	C10—C9	1.419 (3)
C2—C3	1.361 (3)	C9—C8	1.357 (3)
C2—H2	0.9300	C9—H9	0.9300
C3—H3	0.9300	C11—H11	0.9300
C5—H5	0.9300	C8—H8	0.9300
			
C1—N1—C5	123.42 (16)	C7—N7—C11	123.49 (17)
C1—N1—H1	118.3	C7—N7—H7	118.3
C5—N1—H1	118.3	C11—N7—H7	118.3
N4—N3—C6	109.55 (16)	N10—N9—C12	109.34 (16)
N4—N3—H3A	125.2	N10—N9—H9A	125.3
C6—N3—H3A	125.2	C12—N9—H9A	125.3
N2—C1—N1	119.62 (17)	C7—N8—H8A	120.0
N2—C1—C2	122.91 (17)	C7—N8—H8B	120.0
N1—C1—C2	117.47 (16)	H8A—N8—H8B	120.0
C1—N2—H2A	120.0	N8—C7—N7	119.82 (18)
C1—N2—H2B	120.0	N8—C7—C8	122.80 (18)
H2A—N2—H2B	120.0	N7—C7—C8	117.36 (17)
C6—N6—N5	105.81 (16)	N11—N10—N9	106.13 (15)
N5—N4—N3	105.89 (15)	C12—N12—N11	105.95 (16)
N6—C6—N3	107.67 (16)	N12—C12—N9	107.80 (16)
N6—C6—C4	126.56 (17)	N12—C12—C10	126.32 (17)
N3—C6—C4	125.76 (17)	N9—C12—C10	125.87 (17)
N4—N5—N6	111.08 (16)	N10—N11—N12	110.78 (16)
C5—C4—C3	117.77 (17)	C11—C10—C9	118.05 (17)
C5—C4—C6	120.74 (17)	C11—C10—C12	120.76 (17)
C3—C4—C6	121.49 (16)	C9—C10—C12	121.19 (17)
C3—C2—C1	119.91 (17)	C8—C9—C10	120.89 (17)
C3—C2—H2	120.0	C8—C9—H9	119.6
C1—C2—H2	120.0	C10—C9—H9	119.6
C2—C3—C4	120.90 (17)	N7—C11—C10	120.20 (17)
C2—C3—H3	119.5	N7—C11—H11	119.9
C4—C3—H3	119.5	C10—C11—H11	119.9
N1—C5—C4	120.49 (17)	C9—C8—C7	119.93 (17)
N1—C5—H5	119.8	C9—C8—H8	120.0
C4—C5—H5	119.8	C7—C8—H8	120.0
			
C5—N1—C1—N2	177.74 (19)	C11—N7—C7—N8	176.37 (19)
C5—N1—C1—C2	−1.8 (3)	C11—N7—C7—C8	−1.9 (3)
C6—N3—N4—N5	0.0 (2)	C12—N9—N10—N11	−0.2 (2)
N5—N6—C6—N3	0.1 (2)	N11—N12—C12—N9	−0.2 (2)
N5—N6—C6—C4	179.20 (18)	N11—N12—C12—C10	179.35 (18)
N4—N3—C6—N6	0.0 (2)	N10—N9—C12—N12	0.3 (2)
N4—N3—C6—C4	−179.15 (19)	N10—N9—C12—C10	−179.30 (18)
N3—N4—N5—N6	0.1 (2)	N9—N10—N11—N12	0.1 (2)
C6—N6—N5—N4	−0.1 (2)	C12—N12—N11—N10	0.1 (2)
N6—C6—C4—C5	175.7 (2)	N12—C12—C10—C11	−173.8 (2)
N3—C6—C4—C5	−5.4 (3)	N9—C12—C10—C11	5.6 (3)
N6—C6—C4—C3	−4.9 (3)	N12—C12—C10—C9	5.7 (3)
N3—C6—C4—C3	173.99 (19)	N9—C12—C10—C9	−174.82 (19)
N2—C1—C2—C3	−178.97 (19)	C11—C10—C9—C8	−1.1 (3)
N1—C1—C2—C3	0.6 (3)	C12—C10—C9—C8	179.35 (18)
C1—C2—C3—C4	0.9 (3)	C7—N7—C11—C10	−0.6 (3)
C5—C4—C3—C2	−1.2 (3)	C9—C10—C11—N7	2.1 (3)
C6—C4—C3—C2	179.48 (19)	C12—C10—C11—N7	−178.31 (18)
C1—N1—C5—C4	1.6 (3)	C10—C9—C8—C7	−1.4 (3)
C3—C4—C5—N1	0.0 (3)	N8—C7—C8—C9	−175.3 (2)
C6—C4—C5—N1	179.36 (18)	N7—C7—C8—C9	2.9 (3)

### Hydrogen-bond geometry (Å, °)

**Table:**

*D*—H···*A*	*D*—H	H···*A*	*D*···*A*	*D*—H···*A*
N1—H1···N5^i^	0.86	2.59	3.317 (2)	143
N1—H1···N6^i^	0.86	2.04	2.894 (2)	171
N2—H2A···N5^i^	0.86	2.15	2.972 (3)	160
N7—H7···N11^ii^	0.86	2.57	3.304 (2)	144
N7—H7···N12^ii^	0.86	2.06	2.913 (2)	169
N8—H8A···N11^ii^	0.86	2.20	3.024 (3)	160
N2—H2B···Cl1^iii^	0.86	2.36	3.2187 (18)	177
N3—H3A···Cl2^i^	0.86	2.17	3.0047 (19)	165
N8—H8B···Cl2^iv^	0.86	2.39	3.2432 (18)	172
N9—H9A···Cl1^v^	0.86	2.18	3.0031 (18)	160

Symmetry codes: (i) −*x*+1, *y*+1/2, −*z*+1/2; (ii) −*x*+2, *y*+1/2, −*z*+1/2; (iii) *x*, −*y*+3/2, *z*−1/2; (iv) *x*, −*y*+1/2, *z*+1/2; (v) −*x*+2, *y*−1/2, −*z*+1/2.
